# Micro/Nano Energy Storage Devices Based on Composite Electrode Materials

**DOI:** 10.3390/nano12132202

**Published:** 2022-06-27

**Authors:** Yanqi Niu, Deyong Shang, Zhanping Li

**Affiliations:** 1 School of Mechanical, Electronic & Information Engineering, China University of Mining and Technology (Beijing), Beijing 100083, China; sdycumtb@163.com (D.S.); ky202110@cumtb.edu.cn (Z.L.); 2 Institute of Intelligent Mining & Robotics, China University of Mining and Technology (Beijing), Beijing 100083, China; 3 Key Laboratory of Intelligent Mining and Robotics, Ministry of Emergency Management, Beijing 100083, China

**Keywords:** α-Fe_2_O_3_@MnO_2_, electrode materials, electrochemical performance, flexibility

## Abstract

It is vital to improve the electrochemical performance of negative materials for energy storage devices. The synergistic effect between the composites can improve the total performance. In this work, we prepare α-Fe_2_O_3_@MnO_2_ on carbon cloth through hydrothermal strategies and subsequent electrochemical deposition. The α-Fe_2_O_3_@MnO_2_ hybrid structure benefits electron transfer efficiency and avoids the rapid decay of capacitance caused by volume expansion. The specific capacitance of the as-obtained product is 615 mF cm^−2^ at 2 mA cm^−2^. Moreover, a flexible supercapacitor presents an energy density of 0.102 mWh cm^−3^ at 4.2 W cm^−2^. Bending tests of the device at different angles show excellent mechanical flexibility.

## 1. Introduction

Supercapacitors (SCs) have attracted much attention from researchers as an innovative type of energy storage device [1,2,3,4]. Compared with traditional capacitors, SCs shows the advantages of superior cycle stability, outstanding power density and fast charging/discharging [5,6,7]. Recently, electronic devices have progressively high requirements for long-term endurance. However, SCs is severely limited with low energy density [8,9,10]. According to the present research results, one of the most valid ways to settle this issue is to increase the specific capacity of electrode [11]. Therefore, designing electrodes with high specific capacitance is the primary task to broaden the application range of SCs.

Currently, the research on positive and negative materials is unevenly developed and research on negative electrodes is relatively little, which makes it difficult to increase the energy density of SCs. Commonly used negative materials are carbon (AC, CNTs and rGO), transition metal oxides (such as Fe_3_O_4_, α-Fe_2_O_3_, MoO_3_ and Mn_3_O_4_) and a small amount of metal nitride [12,13,14,15,16,17]. Among them, α-Fe_2_O_3_ is considered to have the highest potential and is the most widely used anode material, because of its high redox activity, large theoretical specific capacitance and environmental protection [18]. Nonetheless, the weak conductivity of α-Fe_2_O_3_ electrodes leads low practical specific capacitance and poor electrochemical stability [19,20]. Manganese dioxide (MnO_2_) has gained extensive attention in the construction of supercapacitors due to its high oxidation activity [21]. At present, preparing nanocomposite materials utilizing the synergistic effect of two materials not only promotes redox reactions, but also enhance device energy density [22]. Co_3_O_4_@MnO_2_, SnO_2_@MnO_2,_ ZnO@MnO_2_, CuO@MnO_2_ and α-Fe_2_O_3_@MnO_2_ nanostructures were compounded to achieve both excellent cyclic stability and high capacitance [23,24,25,26].

Seol et al. prepared two types of SCs (EDLC and PC) using activated carbon and graphene/Mn_3_O_4_ nanocomposite. The performance degradation of EDLC was negligible after 100,000 cycles, while PC was less than 10% after 25,000 cycles [27]. Both devices demonstrate excellent cyclic stability and durability. Sarkar et al. fabricated α-Fe_2_O_3_/MnO_2_ nano-heterostructure with a specific capacitance of 750 mFcm^−2^ at 2 mV s^−1^ [28]. However, in practice, these composites, because of loose contact, might impact their electrochemical performance. Thus, it is necessary to construction α-Fe_2_O_3_-based materials with unique nanostructures and excellent electrochemical performance. By combining two materials with high oxidative activity, the synthesis of ordered nanostructures will help to construct electrode materials with excellent specific capacitance. The main objective of our research is that by compounding nanomaterials, the advantages of both can be fully exploited and the electrochemical performance can be effectively enhanced.

Herein, we synthesized α-Fe_2_O_3_ nanorods structures through a hydrothermal route. Then, a MnO_2_ film is coated on α-Fe_2_O_3_ surface by subsequent electrochemical deposition. When utilized as negative material for SCs, α-Fe_2_O_3_@MnO_2_ electrode shows a specific capacitance of 615 mF cm^−2^ at 2 mA cm^−2^. After 10,000 cycles, it maintains 92.3% of the initial capacitance. Finally, a flexible supercapacitor possesses the maximum energy density is 0.102 mWh cm^−3^ at 4.2 W cm^−2^. The results under different angles bending tests demonstrated that the device possesses excellent mechanical flexibility.

## 2. Experimental Section

### Material Preparation

The α-Fe_2_O_3_ sample was synthesized via a hydrothermal method. In total, 0.808 g Fe(NO_3_)_3_·9H_2_O, 0.2841 g Na_2_SO_4_ and 0.5 g PVP were dissolved into 45 mL deionized water. Then, a clean carbon cloth (2.5 × 2.5 cm^2^) and the above mixed solution was transferred into an 80 mL autoclave and kept 110 °C for 9 h. Finally, the as-synthesized samples were annealed at 350 °C for 2 h (2 °C min^−1^). An α-Fe_2_O_3_@MnO_2_ sample was prepared by subsequent electrochemical deposition. In total, 2.4509 g C_4_H_6_MnO_4_·4H_2_O and 1.4204 g Na_2_SO_4_ was used as electrolyte. The α-Fe_2_O_3_ product was used as the working electrode, Ag/AgCl as the reference electrode and Pt foil as the counter one, with deposition at 1 V constant potential for 30 s. The NiCo_2_S_4_ sample was prepared from a homogeneous solution of 0.4 g Ni(NO_3_)_2_·6H_2_O, 1 g Co(NO_3_)_2_·6H_2_O, 0.5 g urea, 0.1 g NH_4_F and 60 mL deionized water, heated with nickel foam at 140 °C for 12 h. It was then combined with 0.5 g Na_2_S·9H_2_O and 60 mL deionized water at 140 °C for 6 h. α-Fe_2_O_3_, α-Fe_2_O_3_@MnO_2_ and NiCo_2_S_4_ mass loading is 2, 2.3 and 1.2 mg cm^−2^, respectively.

A supercapacitor was assembled with PVA-KOH gel as the electrolyte, NiCo_2_S_4_ as the positive electrode and α-Fe_2_O_3_@MnO_2_ as the negative electrode. The preparation process of PVA-KOH gel electrolyte is as follows: stir 2 g KOH with 2 mL distilled water, mix well and set aside for later use. In a 20 mL beaker, add 2 g polyvinyl alcohol (PVA) and 20 mL deionized water, and stir at 80 °C until transparent. Finally, drop the KOH solution into the PVA solution at a constant speed, and stir at a constant temperature until it becomes a clear and transparent gel.

The crystal structure and the elemental compositions of the products were investigated by an X-ray diffractometer (XRD, Shimadzu-7000, Kyoto, Japan, CuKα, 40 kV) and X-ray photoelectron spectrometer (XPS, Amsterdam, Holland,). The morphology and microstructure of the sample is characterized by scanning electron microscope (SEM, Gemini 300-71-31, Berlin, Germany).

In a three-electrode system, the as-prepared electrode was measured through an electrochemical workstation (Shanghai Chenhua). Electrochemical performance methods include cyclic voltammetry (CV), galvanostatic charge-discharge (GCD) and electrochemical impedance spectroscopy (EIS). The as-synthesized materials were used as the working electrode, Pt foil as the counter electrode and Ag/AgCl as the reference electrode.

## 3. Results and Discussion

**Figure 1:**
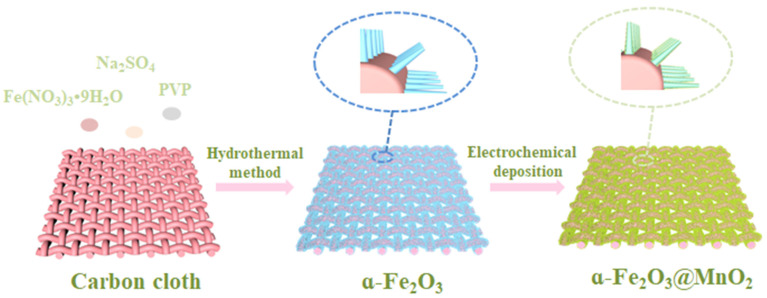
Synthesis schematic of the products.

Figure 1 presents the growth process of α-Fe_2_O_3_@MnO_2_ products on carbon cloth. Firstly, α-Fe_2_O_3_ nanorods are obtained via a facile hydrothermal approach. Afterwards, a layer of MnO_2_ film is deposited by subsequent electrochemical deposition on the nanorod-shaped α-Fe_2_O_3_ surface.

**Figure 2:**
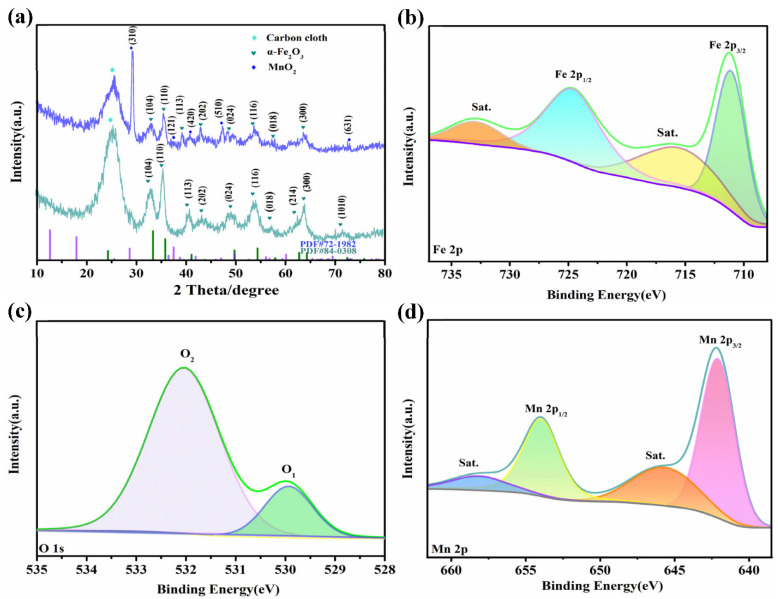
Structural characterization using (**a**) XRD patterns and (**b**–**d**) XPS spectra.

First, the crystal structure of the obtained product is studied by XRD. Figure 2a shows the XRD patterns of α-Fe_2_O_3_ and α-Fe_2_O_3_@MnO_2_ composites. A typical peak of the carbon cloth can be clearly observed. The peaks at 2θ values of 33.4°, 35.8°, 49.7°, 54.4°, 64.3° and 72.4° can be indexed to (104), (110), (024), (116), (300) and (1010) planes of α-Fe_2_O_3_ phases, respectively (PDF No. 84-0308). Those at 28.7°, 37.6°, 41.1°, 47.2° and 72.6° match well with (310), (121), (420), (510) and (631) planes of MnO_2_ (PDF No. 72-1982). The shape and sharpness of the diffraction peaks in figure reveal that the products possess high crystallinity.

Then, XPS is used to investigate the α-Fe_2_O_3_@MnO_2_ materials surface element composition. In Fe 2p spectra, the characteristic peaks of Fe 2p_3/2_ and Fe 2p_1/2_ at 711.2 eV and 724.8 eV, respectively (Figure 2b). Additionally, two shake-up satellite peaks (Sat.) at 716 eV and 732.9 eV are determined. This indicates that Fe^3+^ exists in composite product [29]. Figure 2c depicts the two main peaks of O 1s spectra located at 529.9 eV and 532 eV [30]. Binding energies at 529.9 eV, labeled as O_1_, denote metal oxygen [31]. Another O_2_ peak located at 532 eV is due to some degree of hydrolysis on the product surface [32]. For Mn 2p spectra (Figure 2d), four peaks at 642.2 eV, 645.8 eV, 653.9 eV and 658.1 eV are from Mn 2p_3/2_, Sat., Mn 2p_1/2_ and Sat., respectively [33].

**Figure 3:**
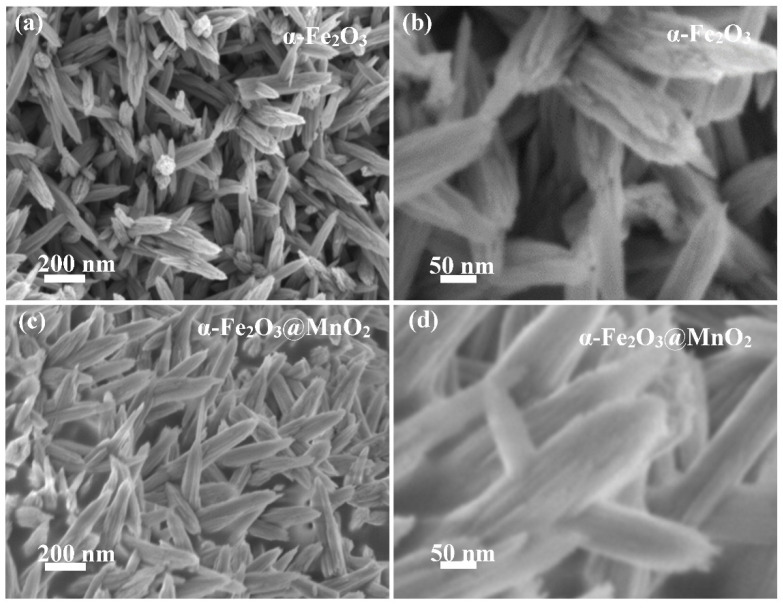
SEM images of the samples. (**a**,**c**) single materials (**b**,**d**) conposite materials.

Figure 3a indicates that α-Fe_2_O_3_ shows a short rod-like structure. In addition, it can be found that many nanorods homogeneously grown on carbon cloth with uniform size and shape, and the cross-section of nanorods is rough. The high magnification image (Figure 3b) shows the as-synthesized products average length is 100 nm. Figure 3c presents a thin MnO_2_ film covers α-Fe_2_O_3_, and still maintains the shape of nanorods. From Figure 3d, the cross-section of α-Fe_2_O_3_@MnO_2_ nanorods becomes smooth.

**Figure 4:**
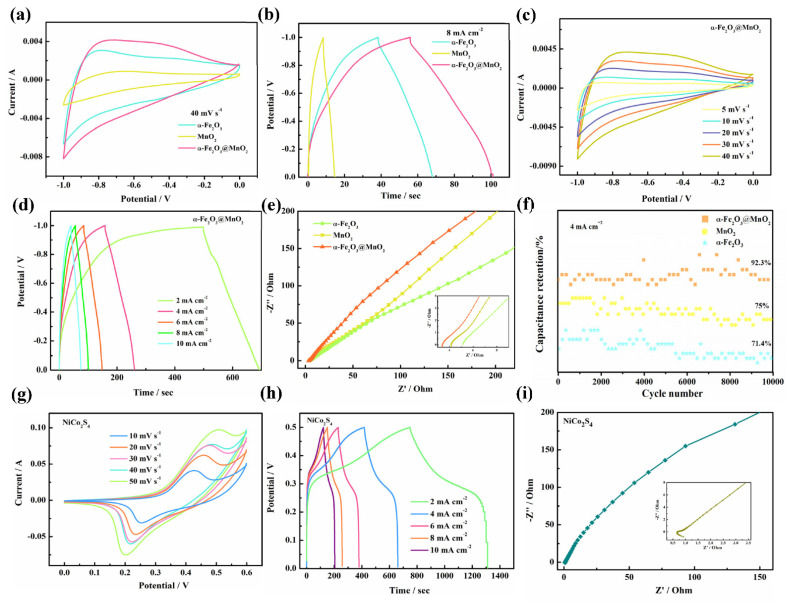
Electrochemical performance. (**a**) CV curves. (**b**) GCD curves. (**c**) CV curves of α-Fe_2_O_3_@MnO_2_. (**d**) GCD curves of α-Fe_2_O_3_@MnO_2_. (**e**) Nyquist plots. (**f**) Cycling performance at 4 mA cm^−2^. (**g**) NiCo_2_S_4_ CV curves. (**h**) NiCo_2_S_4_ GCD curves. (**i**) NiCo_2_S_4_ Nyquist plots.

Next, we analyzed several as-obtained electrode electrochemical performances by CV, GCD and EIS. Figure 4a shows CV curves of α-Fe_2_O_3_, MnO_2_ and α-Fe_2_O_3_@MnO_2_ materials. Evidently, α-Fe_2_O_3_@MnO_2_ delivers a large CV area in −1–0 V, reflecting its good energy storage effect in this range. At 8 mA cm^−2^ (Figure 4b), the GCD curves obvious that α-Fe_2_O_3_@MnO_2_ product with long discharge times, which can be correlative to the synergistic effect between α-Fe_2_O_3_ and MnO_2_ materials. Figure 4c presents CV curves of α-Fe_2_O_3_@MnO_2_ from 5 to 40 mV s^−1^. The shape of CV curves almost the same as the scan rate increased, indicating excellent reversibility of electrode. In Figure 4d, the GCD curves of α-Fe_2_O_3_@MnO_2_ materials are measured from 2 to 10 mA cm^−2^. Areal capacitance (C_a_) is obtained by GCD, and the equation is shown below:

Ca = I∫Vdt/V(1)

In Equation (1), I is current density, ∫ Vdt stands for the integral area of discharge curve and V is the constant discharge voltage range (V). The α-Fe_2_O_3_@MnO_2_ electrode delivers 615 mF cm^−2^ specific capacitance at 2 mA cm^−2^

EIS is a significant factor in assessing the electrochemical kinetics of products. The sample is tested over a frequency range of 0.01 Hz to 100 kHz (Figure 4e). In the low frequency region, the slope of the straight line shows the ion diffusion resistance. Among the three samples, α-Fe_2_O_3_@MnO_2_ sample presents the largest slope, which expresses fast diffusion of ions in electrolyte [34]. The intersection with the real axis represents the equivalent resistance (Rs) [35]. α-Fe_2_O_3_, MnO_2_ and α-Fe_2_O_3_@MnO_2_ electrodes Rs value is 5.1 Ω, 4.1 Ω and 3.3 Ω, respectively. According to above analysis, α-Fe_2_O_3_@MnO_2_ shows the largest slope and smallest Rs, so the conductivity of composite material is better than α-Fe_2_O_3_ and MnO_2_.

At the end, the cyclic stability is investigated at 4 mA cm^−2^. Figure 4f indicates that the capacitance of α-Fe_2_O_3_@MnO_2_ is only reduced by 7.7% after 10,000 cycles, while α-Fe_2_O_3_ and MnO_2_ products present only 71.4% and 75% of the initial capacitance. This phenomenon is due to the MnO_2_ film covering the α-Fe_2_O_3_ nanorods, which can help alleviate the volume expansion during long cycle measurements. Similarly, the positive NiCo_2_S_4_ is also studied by the same methods. Figure 4g presents the CV curves of NiCo_2_S_4_ sample. Redox peaks and shapes, confirming its pseudocapacitive material. Five symmetrical GCD curves shows an obvious platform (Figure 4h), which indicates their Faradaic redox behavior [36]. At 2 mA cm^−^^2^, the specific capacitance is 720.8 mF cm^−^^2^. Nyquist plots of NiCo_2_S_4_ products are shown in Figure 4i; the value of Rs is 0.9 Ω.

**Figure 5:**
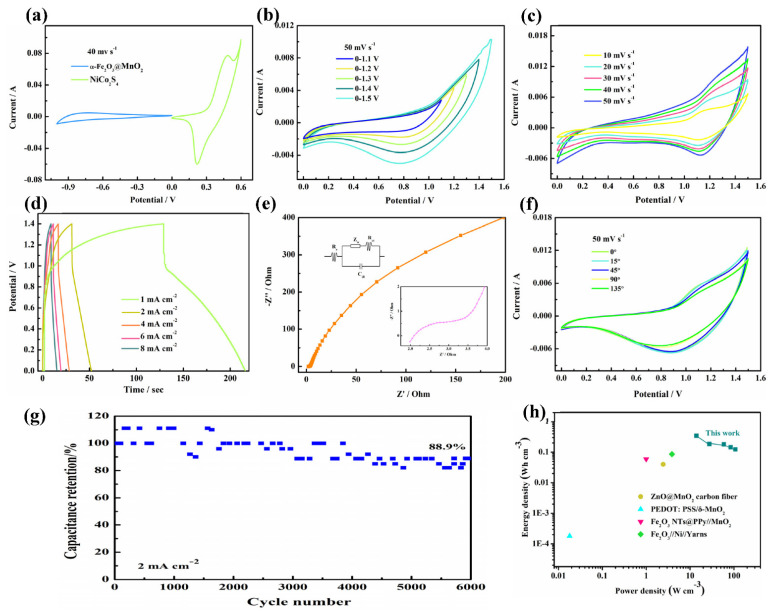
(**a**) CV curves of the α-Fe_2_O_3_@MnO_2_ and NiCo_2_S_4_ electrode at 40 mV s^−1^. (**b**) CV curves in different potential windows at 50 mV s^−1^. (**c**) CV curves. (**d**) GCD curves. (**e**) EIS. (**f**) CV curves at different bending angles. (**g**) cycling performance at 2 mA cm^−2^. (**h**) Ragone plot.

To further explore the α-Fe_2_O_3_@MnO_2_ electrodes for practical applications, a flexible supercapacitor is assembled. From Figure 5a, the voltage windows of α-Fe_2_O_3_@MnO_2_ and NiCo_2_S_4_ are −1–0 V and 0–0.6 V, respectively. Figure 5b shows CV curves from 1.1 V to 1.5 V with a sweep rate of 100 mV s^−1^, demonstrating the device can maintain operate stably within 1.5 V. It can be seen that with the decrease of voltage, the area becomes small. Figure 5c depicts all CV curves at different scan rates keep similar shapes, revealing outstanding rate performance of device. GCD curves from 1 to 8 mA cm^−2^ possess the same charging and discharging time (Figure 5d). The specific capacitance of the device at 1 mA cm^−^^2^ is 37.8 mF cm^−^^2^ and it still delivers 15.6 mF cm^−^^2^ at 8 mA cm^−^^2^. The equivalent resistance value of the device is 1.9 Ω, as shown in Figure 5e.

At present, electronic devices are developing towards wearable, which puts forward higher requirements for the mechanical flexibility of supercapacitors [37]. We twisted the device and then examined it by cyclic voltammetry (Figure 5f). While device is folded at 15°, 45°, 90° and 135°, the shape sustains virtually unchanged, demonstrating its superior mechanical stability. Figure 5g illustrates that the device maintains 88.9% capacitance retention after 6000 cycles. Figure 5h is the Ragone diagram of α-Fe_2_O_3_@MnO_2_//NiCo_2_S_4_. The capacitor values of energy density (E) and power density (P) can be derived based on the Equations (2) and (3):

E = 1/2 × C_a_ × V^2^ (2)

P = 3600 × E/Δt(3)

where C_a_ stands for the areal capacitance of the capacitor, V represent the discharge voltage and Δt is the discharge time. At 1 mA cm^−2^, the energy density of device is 0.102 mWh cm^−3^ at 4.2 W cm^−2^. This is better than some previously reported materials [38,39,40,41] (Table 1).

α-Fe_2_O_3_@MnO_2_ delivers excellent performance, which can be explained by the following reasons: (a) Nanostructure uniformly covered on the carbon cloth, which provides outstanding electrical conductivity and flexibility; (b) With α-Fe_2_O_3_ as a strong mechanical support and MnO_2_ as an outer layer, this structure not only protects the morphological structure, but also provides many active sites; (c) The composite utilizes the synergistic effect of α-Fe_2_O_3_ and MnO_2_, so that electrode processes high capacitance and low resistance.

## 4. Conclusions

In this manuscript, α-Fe_2_O_3_@MnO_2_ nanorods are synthesized through a hydrothermal route and subsequent electrochemical deposition. By combining two oxides of α-Fe_2_O_3_ and MnO_2_, it is favorable to accelerate the electron transport and the oxidation reaction. The synergistic effect between two materials improves electrochemical performance for negative electrode. MnO_2_ film, after electrodeposition, affects the performance of the electrode material, and the full use of the active area of the film increases, which increases the capacitance of the electrode material. XPS results show that the material processes abundant redox valence states. α-Fe_2_O_3_@MnO_2_ sample presents high specific capacitance and excellent cycling stability. Furthermore, the as-assembled capacitors still show outstanding electrochemical performance and mechanical stability. Therefore, it provides an alternative method for constructing supercapacitor negative materials with higher specific capacitance.

## Figures and Tables

**Table 1:** Electrochemical performance of various devices.

Supercapacitor	Capacitance	Energy Density (mWh cm^−3^)	Power Density (W cm^−2^)	Capacitance Retention	Ref.
PEDOT: PSS/δ-MnO_2_	2.4 F cm^−3^	0.018	0.018	88%	[38]
Fe_2_O_3_NTs@PPy//MnO_2_	-	0.0594	1	92%	[39]
ZnO@MnO_2_	26 mF cm^−2^	0.04	2.44	87.5%	[40]
Fe_2_O_3_//Ni/Yarns	0.67 F cm^−3^	0.086	3.87	87.1%	[41]
α-Fe_2_O_3_@MnO_2_//NiCo_2_S_4_	37.8 mF cm^−2^	0.102	4.2	88.9%	this work

## Data Availability

Not applicable.
